# The Late Dr. Frank H. Potter

**Published:** 1891-10

**Authors:** 


### BUFFALO MEDICAL AND SURGICAL JOURNAL

A MONTHLY REVIEW OF MEDICINE AND SURGERY.

*EDITORS:*

THOMAS LOTHROP, M. D. - - WM. WARREN POTTER, M. D.

All communications, whether of a literary or business character, should be addressed to the managing editor : 284 FRANKLIN STREET, BUFFALO, N. Y.

VOL. XXXI. OCTOBER, 1891. No. 3.

### THE LATE DR. FRANK H. POTTER.

THE untimely death of our late associate editor, Dr. Frank Hamilton Potter, has called forth much gratifying eulogium from the newspapers of Buffalo, as well as the medical journals throughout the country. These comments have been of such a nature as to deeply touch the hearts of the two families that have been so cruelly invaded and bereft of a son, husband, and father.

Especially has the managing editor of the JOURNAL, the father of the deceased, felt a deep and grateful sense of appreciation of the kindness manifested by his many friends throughout the country for their sympathizing words, both written and printed. He has been lifted out of a dark and dangerous despondency by these very tributes to his accomplished son, while the shower of personal letters he has also received, that have breathed tender and affectionate appreciation of both father and son, have been a tower of strength to his afflicted soul.

We feel it only an act of justice to all concerned to reprint these newspaper and journalistic articles, that they may be grouped for preservation to the memory of our lamented colleague.

T. L.

---

[Buffalo Morning Express, July 17, 1891.]

### OBITUARY.

#### DEATH OF FRANK H. POTTER, M. D

The report of the fatal illness of Dr. Frank H. Potter will prepare the public for the further announcement of his death, which occurred at his home on Franklin street at seven o'clock last evening. The blow has fallen swiftly. Early last week he was only slightly ill, and rode out on his wheel. On Saturday last he submitted to an operation for the removal of a perityphlitic abscess. Following the operation vomiting set in, which was not controlled for thirty-six hours, and the prostration resulting carried him past recovery.

Dr. Potter was the only son of Dr. W. W. Potter, of this city, and was born at Cowlesville, Wyoming county, on January 8, 1860. He was, therefore, just past his 31st year. As a practitioner and student and teacher he stood high in his profession. He graduated from the Buffalo Medical College in 1882, having already been connected for two years with the Rochester City Hospital. In 1883 he was appointed clinical assistant to the chair of surgery in Niagara Medical College. He was also lecturer on anatomy, and had given lectures on laryngology in the same college.

He was a member of the surgical staff of the Sisters of Charity and Emergency hospitals, and was lately appointed clinical professor of laryngology in the Buffalo Medical College. He was nominated by the Council a member of the American Laryngological Association for this year. He was secretary of the section of laryngology in the American Medical Association, member of pathological and obstetric societies of the city, of the Buffalo Medical and Surgical Association, the Erie County Medical Society, and of the New York State Medical Society, besides various medical clubs.

On June 11, 1887, he married Eva, daughter of Mr. L. G. Sellstedt, who survives him, together with two sons, one three years and the other four months old. All who knew Dr. Potter will regret his early death. Whether as physician, teacher, or friend, he was a man greatly esteemed and beloved, and he will be deeply missed both by the profession and the community.

The funeral will take place from the residence of Dr. W. W. Potter, No. 284 Franklin street, at 3.30 o'clock tomorrow afternoon.

---

[Buffalo Courier, Friday, July 17, 1891.]

### OBITUARY.

### DR. FRANK HAMILTON POTTER.

Dr. Frank Hamilton Potter, of No. 273 Franklin street, died at 7 o'clock last night, after an illness of ten days' duration.

He was the only son of Dr. William Warren Potter, of No. 284 Franklin street, and was born in Cowlesville, Wyoming county, January 8 1860, being in his 32d year at the time of his death. In 1882, when he was 22 years of age, he was graduated from the Buffalo Medical College. Prior to this time he had served as resident physician in the Rochester City Hospital for two years. In 1883, at the organization of the Niagara University, he was appointed clinical assistant to the chair of surgery, and subsequently held the position of lecturer in anatomy and laryngology. He at one time was a member of the surgical staff of the Sisters of Charity and Emergency hospitals. He first began the practice of general medicine, which he continued for a time, but afterward gave it up for the special field of laryngology, for which he had fitted himself in the schools of both this country and Europe. But recently he was appointed clinical professor of laryngology of the University of Buffalo. He was also associate editor of the BUFFALO MEDICAL AND SURGICAL JOURNAL with his father. He was a member of numerous medical societies, among which were the Buffalo Medical and Surgical Association, Erie County Medical Society, Medical Society of the State of New York, Buffalo Pathological Society, and Obstetrical Society. He was also a member ot the Saturn and Thursday clubs. During the year 1890 he was secretary of the laryngological section of the American Medical Association, and he was recently chosen by the Council of the American Laryngological Association for membership in that organization.

In political life he had taken a small part, having served two terms as civil service commissioner. He was removed from that office by Mayor Becker along with Mr. Loomis, but was re-instated when Mayor Bishop was elected. This office he resigned about a year ago on account of other business duties.

In 1887 he was married to Eva, daughter of Lars G. Sellstedt. To them two children were born, both of whom, with his wife, survive him. He was an active and hard-working member of his profession, and had until recently enjoyed the best of health. A week ago last Sunday he was taken ill, but was not compelled to take to his bed until Wednesday. It was then found that he was suffering from an enlargement of one side of the abdomen. On Saturday a surgical operation was performed, at which time nearly six ounces of pus were taken from the region of the colon. Blood poisoning had set in, and he failed rapidly, dying last night.

The funeral will be held on Saturday afternoon at 3 o'clock.

[Editorial in the Courier, July 17th.]

### THE LATE DR. F. H. POTTER.

The death of Dr. Frank H. Potter deprives the community not only of a skilled physician, who gave every promise of achieving eminence in a special branch of his profession, but also, which makes his loss even more lamentable, of a man who stood as the type of a good citizen. For, in spite of his professional ability and enthusiasm, he was unwilling to sink the man in the physician. He took a zealous interest in public affairs, and was ready to give time and labor to the advancement of reforms, not as a partisan, but as a lover of good government and progressive politics. He cultivated every side of a well-rounded nature. He had a fine literary taste, and enjoyed the more elevated forms of verse and prose. He had a clear understanding of mechanical principles, and keenly appreciated the relative merits of rival forms of machinery. He was a delightful associate, interested in all subjects, and well informed in most, with a delicate sense of the humorous and picturesque in life, and withal of a singularly modest, gentle, and pure character. Those who knew Dr. Potter at all, liked him, and those who knew him best loved him.

---

[Buffalo Commercial, Friday, July 17, 1891.]

### DEATH OF FRANK H. POTTER.

### THE YOUNG PHYSICIAN PASSED AWAY LAST EVENING — SKETCH OF HIS LIFE.

Dr. Frank Hamilton Potter died at 7 o'clock last evening at his residence, No. 273 Franklin street, of the peculiar disease known as appen. dicitis, which resulted in blood poisoning. As stated in the *Commercial*, an abscess caused by the lodging of a seed or other foreign substance in the little sac known as the vermiform appendix. An operation was recently performed, but blood poisoning had already taken place, and he sank rapidly. It was known yesterday that he could not live. "He cannot live through the day," was the word from Dr. Parmenter, who remained with him almost constantly during the last days of his life. Towards evening there was a slight rally in the condition of the patient, raising a feeble hope, but it proved to be only a flicker of the light before darkness came.

Dr. Potter was one of the best known of the younger physicians of this city, and also one of the best liked. He was the sort of a man who would be picked out instinctively as one who could be trusted, who would be a true friend. That judgment would have been correct.

He was born on January 8, 1860, in Cowlesville, Wyoming county, and was the only son of Dr. William W. Potter, now of No. 284 Franklin street, this city. He graduated from the Buffalo Medical College in 1882, when but 22 years of age. By this time he was well schooled in his profession, as he had already served as resident physician in the Rochester City Hospital for two years. When Niagara University was organized, in 1883, Dr. Potter was appointed clinical assistant to the chair of surgery, and later on took the position of lecturer in anatomy and laryngology. He soon forsook general practice to make a specialty of throat diseases. He was very skilful in his specialty, having devoted much study to it in this country and Europe. His skill in this particular received fitting acknowledgment quite recently when he was appointed clinical professor of laryngology at the University of Buffalo.

Some years ago he was on the surgical staff of the Emergency and Sisters' hospitals. He was a writer of considerable ability, as demonstrated by his work as associate editor with his father, of the BUFFALO MEDICAL AND SURGICAL JOURNAL. He was a member of the Buffalo Medical and Surgical Association, Erie County Medical Society, Medical Society of the State of New York, Buffalo Pathological Society, and Obstetrical Society. During 1890 he was secretary of the laryngological section of the American Medical Association, and was recently chosen by the Council for membership in the American Laryngological Association. The social and literary side of his character found expression in the Saturn and Thursday clubs, in which he held membership.

Dr. Potter served two terms as civil service commissioner. He resigned the office about a year ago. He was married in 1887 to Eva Sellstedt, daughter of L. G. Sellstedt, the well-known artist. His wife and two children survive him.

[The Buffalo Commercial, July 18th.]

DR. POTTER'S FUNERAL.

The funeral of the late Dr. Frank Hamilton Potter was held this afternoon at 3.30, at the residence of his father, 284 Franklin street. The services were conducted by the Rev. Mr. Ferguson, rector of St. James' Church, Syracuse, and Rev. H. G. Lord, pastor of the West-Side Presbyterian Church. After the services were concluded, the body was taken to the Buffalo crematory for incineration, The bearers were: Dr. James W. Putnam, Dr. John Parmenter, Dr. John O. Roe, Mr. Seward A. Simons, Mr. E. R. Rice, Mr. F. F. Williams, Mr. Carlton Sprague, Mr. F. J. Shepard.

[The Buffalo Commercial editorial column.]

The death of Dr. Frank Hamilton Potter, of this city, at the early age of 31, is a very grievous affliction to his friends, and a real loss to the community. He was a well-educated physician, with a growing fame and practice. He was a companionable man, of varied tastes and sympathies, as the active part he took in public affairs and in literary and social clubs testified. His future seemed as bright and promising as that of any of the younger professional men in Buffalo. His taking-off seems untimely ; but perhaps it is wiser to regard it as the passing on to an earlier fulfillment of the promise of his youthful excellence.

---

[Editorial in the Times, Friday evening, July 17, 1891.]

The *Times* said in its editorial columns :

. . . . . . . . . . . . . . . . . .

Dr. Potter was a surgeon of unusual skill in his special line of work, and active in social clubs and orders. He had written much on professional subjects, and took an unusually active interest in public affairs. The deepest sorrow will be felt by all who knew him, that one whose life was so full of promise for good should be called away at the very threshold of a distinguished career.

[The Times, Saturday evening, July 18, 1891.]

### IN MEMORY OF DR. POTTER.

### TRIBUTES PAID BY MEMBERS OF THE PROFESSION IN BUFFALO.

Resolutions of respect to the memory of the late Frank H. Potter were passed yesterday afternoon by the faculties of the Niagara and Buffalo medical colleges. A special meeting of the Erie County Medical Society was held in the evening, and a committee consisting of Drs. Bartlett. Abbott, and Rochester submitted the following resolutions, which were adopted :

[These resolutions appear on page 42, August number of the Journal.]

Remarks were made by Drs. Parmenter, Snow, Clark, Putnam, Howe, Cronyn, Ingraham, Pryor, Hubbell, Abbott, and Rochester. The time of the funeral has been changed to 3.30 o'clock this afternoon.

[The Times, Friday evening, July 17, 1891.]

### DEATH OF DR. POTTER.

### HE SUCCUMBS LAST EVENING AFTER A BRIEF ILLNESS.

Dr. Frank Hamilton Potter died at 7 o'clock last evening, at his residence, No. 273 Franklin street, after a ten days' illness.

The news of Dr. Potter's death will be heard with sincere regret by many friends at home and abroad. His illness was known to but a few. Early last week he was slightly indisposed, but still took pleasure in riding out on his wheel. On Saturday it became necessary to perform a surgical operation on him for the removal of a perityphlitic abscess in the region of the head of the colon. About six ounces of pus was removed. Vomiting followed the operation which could not be controlled for thirty-six hours. The resulting prostration, coupled with septicemia, brought about a rapidly fatal termination.

Dr. Potter was the only son of Dr. W. W. Potter, of this city, and was born January 8, 1860, at Cowlesville, Wyoming county. He graduated from the Buffalo Medical College in 1882, and engaged in the practice of general medicine.

Later he prepared himself for the special field of laryngology, both in this country and Europe, and thereafter devoted his entire attention to this specialty. During his medical career he held at various times the positions of clinical assistant to the chair of surgery, lecturer on anatomy, and lecturer on laryngology in the Niagara Medical College. He was at one time a member of the surgical staff of the Sisters of Charity and Emergency hospitals, and had just recently been appointed to the chair of Professor of Laryngology in the Buffalo Medical College. He was associate editor of the BUFFALO MEDICAL AND SURGICAL JOURNAL, a member of the State Medical Society, ex-secretary of the laryngological section of the American Association, and a member of all the local medical societies and other organizations. He also held the office of civil service commissioner for two terms, resigning about a year ago owing to increasing professional business.

In 1887 he married Eva, daughter of Mr. L. G. Sellstedt, who survives him with two sons, one three years and the other four months old.

Dr. Potter was greatly esteemed by all who knew him, either in his professional or private life, and numbered many people at home or abroad among his friends. The funeral will take place from the residence of his father, No. 284 Franklin street, at 3.30 tomorrow afternoon.

---

[The Illustrated Buffalo Express, Sunday, July 26, 1891.]

(With Portrait.)

### FRANK HAMILTON POTTER, M. D.

### AN ABLE YOUNG PHYSICIAN WHOSE DEATH IS DEEPLY MOURNED.

The sudden death of such a man as the late Dr. Frank Hamilton Potter, of this city, which occurred July 16th, in his 32d year, a man whose earlier student life already showed abundant fruits in practice, has brought anguish to his family, deep sorrow to his friends, and genuine regret to those that knew him even slightly.

Dr. Potter was born at Cowlesville, Wyoming county, New York, January 8, 1860, and was the only son of Dr. William Warren Potter, also a prominent member of the medical profession, residing in Buffalo.

The younger Dr. Potter graduated from the Buffalo Medical College in 1882, after having received academical training to fit him for his professional work. Prior to his graduation he served two years as resident physician in the Rochester City Hospital. He was engaged in general practice for several years, during which he served as assistant to the chair of surgery in the Niagara Medical College, as lecturer on anatomy, and, finally, after adopting laryngology as his specialty, he was appointed lecturer on diseases of the nose and throat in that college. He served also as a member of the surgical staff of the Sisters of Charity and the Emergency hospitals. In 1889 he was elected secretary of the laryngological section of the American Medical Association, and lately had been chosen by the Council for membership in the American Laryngological Association. Very recently the faculty of the Medical Department of the University of Buffalo elected him as clinical professor of laryngology in that institution. All these honors attest his general and special acquirements in his chosen field.

He was twice appointed civil service commissioner, resigning in the midst of his second term because of the increase of professional cares. But he was always ready to give of his time and strength to the duties of the citizen. His was not a one-sided development. He was active socially, as a member of the Saturn Club, and was one of the twenty-five members of the Thursday Club, a purely literary society.

No man who has worked thus, even though cut off untimely, has been here in vain. His influence in the community was good, and shall not cease. To his friends he has left a high example, and to those dearer ones a sweet, inspiring memory.

In June, 1887, he married Eva, daughter of Mr. L. G. Sellstedt, the well-known artist of this city, and his widow and two children, one three years and the other four months old, survive him.

---

[From the St. Louis Medical Mirror, Aug. 1, 1891.]

### DR. FRANK HAMILTON POTTER.

It is with extreme sorrow that we learn of the death of Dr. Frank Hamilton Potter, the only son of a devoted father,—and how much is expressed in that sentence? The only son of a doctor, who has been so favorably impressed with the profession of medicine by the example of his father as to desire to follow in his footsteps. Fully alive to the burdens and cares and sorrows and heart-burnings that are connected with the practice of medicine, he must needs be a young man of nerve, must have indeed been impressed favorably by his association with his father, —his partner and chum—in order for him to have been willing to assume the duties connected with the profession for life; but just such a person was Dr. Frank Potter. Not many years' difference between the ages of the two,—just enough to be compatible with the proper laws of physiological reproduction—the two men were all the world to each other. The eminence achieved by Dr. William Warren Potter, the father, served as a stimulus to the son. He worked, and he won, and he achieved a very enviable position in his profession. Not only that, but for one so young, he must have been more than an ordinary man, in his relations to his native place as a citizen. The Buffalo papers were filled with column after column of eulogium referring to his work as a physician, to his sterling integrity as a man, and to his value as a progressive citizen. Hardly an interest in Buffalo but that mourned the death of Dr. Potter. Young and ambitious, full of honors already achieved, what a brilliant future was before him; and yet, in the harness, in the midst of his work taken down suddenly with an obscure trouble which soon developed the necessity of abdominal section,—a suppurating appendicitis was discovered but too late, and a fatal septic peritonitis carried him away.

The young family of Dr. Potter are left to mourn him,—how deeply, can never be told,—but the other family, the elder ones, how great is their sorrow, too! A doting mother, proud of her boy; a strong, able, manly father, still young in years, and ambitious for himself, but more ambitious for his boy, are left almost like shipwrecked sailors. Our hearts go out to them in deepest sorrow. We think of our own boy of four years, one of the idols of our life now, as we look way down the vista of the future, and as the possibilities of such a record come before us, we could feign turn aside and say to ourselves, "Is life worth living?" And yet we believe it is worth living. It is worth living for the work there is in it; and Dr. Potter, Sr., must needs keep his face to the front and his hand to the plow and work, because after all that is the greatest solace in sorrow. He can rest assured that he has the sympathy of the medical profession of the United States.

---

[The Times and Register, Philadelphia, August 1, 1891.]

Dr. Frank H. Potter, only son of Dr. William Warren Potter, of Buffalo, died July 16th, of appendicitis. An operation had been performed, but blood-poisoning set in.

Dr. Potter was but thirty-one years of age, but had already established his reputation as one of the rising men of his profession. At the time of his death he was clinical professor of laryngology in the University of Buffalo, and was associated with his father in the editorship of one of our most valued exchanges—the BUFFALO MEDICAL AND SURGICAL JOURNAL.

We tender our sincere sympathy to his family in their bereavement.

---

[The Canadian Practitioner, Toronto, August 1st.]

Dr. Frank H. Potter, of Buffalo, only son of Dr. W. W. Potter, of the same city, died at his home, July 16th, after an illness of about twelve days, from pericecal abscess. A section was made by Dr. Mordecai Price, of Philadelphia, and about six to eight ounces of pus evacuated. The prospects were good for a time, but he commenced to sink after the fourth day of operation, and death occurred on the following day. He was a young man, *aet.* 31, of unusual promise and most lovable disposition, and his untimely death was a sad blow to his relatives and many friends.

---

[The Cincinnati Lancet-Clinic, August 22, 1891.]

### OBITUARY.

### THE LATE DR. FRANK HAMILTON POTTER.

The medical press is seldom called upon to record a more melancholy and untimely death than that of Dr. Frank Hamilton Potter, which occurred at Buffalo, N. Y., July 16, 1891. Dr. Potter was a young man—not yet thirty-two—but the son and grandson of distinguished physicians. He entered the profession with endowments that speedily gave him recognition and position among the most distinguished of his confrères. After a preliminary education, he became a student in the medical department of the University of Buffalo, from which he received the Doctorate in 1882. It was at once apparent that in graduating, he had received not only his diploma, but that with it he had received inspiration from the spirits of Austin Flint, Sr., and Charles, A. Lee, and Frank Hamilton, and Miner, and others, who, as founders and teachers, had brought fame to his *alma mater*. Within a year afterwards he was elected associate to the chair of surgery in the then recently organized Medical Department of Niagara University, in which school he subsequently and successfully taught anatomy and laryngology, the latter in recent years being his chosen field of practice. But a few months prior to his death he had been invited to, and had accepted the chair of laryngology in the Medical Department of the Buffalo University. He was a member of local and national medical societies, the latter embracing the American Medical and American Laryngological Associations. He was secretary of the Section on Laryngology and Otology of the American Medical Association at Nashville in 1890.

The following list of contributions from his pen shows how enormously he had tilled the field of professional knowledge :

The Proper Use of Ergot in Obstetrical Practice, B. M. & S. J., 1886-8, xxvi., 55-64.

Some General Rules which will Determine the Necessity of an Operation in the Nasal Passages, B. M. & S. J., 1886-7, xxvi., 443-447.

A Curious Effect of Cocaine, B. M. & S. J., 1887-8, xxvii., 210-212.

Tuberculosis of the Nose, Mouth and Pharynx, B. M. & S. J., 1887-8, xxvii., 295-303.

The Galvano-Cautery in the Treatment of Enlarged Tonsils, *Med. News*, Philadelphia, 1888, 255-257.

Notes on the Treatment of Acute Tonsilitis in Children, B. M. & S. J., 87-8, xxvii., 455-458.

Report of a Case of Congenital Bony Occlusion of the Anterior Nares, B. M. & S. J., 1888-9, xxviii., 74-96.

Notes on the Treatment of Acute Coryza, B. M. & S. J., 1888-9, xxviii., 302-307.

The Galvano-Cautery in the Treatment of Enlarged Tonsils, *Trans. Med. Soc. N. Y.*, 1888, 392-397.

Membranous Rhinitis, *Jour. Laryngol.*, London, 1889, III., 89-92.

A Mechanical Nasal Saw, B. M. & S. J., 1888-9, xxviii., 671.

Membranous Rhinitis, *Trans. Med. Soc. N. Y.*, Philadelphia, 1889, 222-228.

The Influence of Oral Irritation in the Production of Diseases of the Upper Air Tract, B. M. & S. J., 1889-90, xxix., 12-17.

The Use of Menthol in Diseases of the Upper Air Passages, *Jour. Amer. Med. Asso.*, Chicago, 1890, xiv., 147-149.

Reflex Asthma Especially of Nasal Origin, B. M. & S. J., 1889-90, xxix., 463-468.

Notes on Nasal Hemorrhage, *Jour. Amer. Med. Asso.*, Chicago, 1890, xv., 467.

Neurasthenia and Nasal Disease, B. M. & S. J., 1890-91, xxx., 337-415.

A Second Note on Croupous Rhinitis, B. M. & S. J., 1890-91, xxx., 525-527.

Dr. Potter leaves a distinguished father, Dr. William Warren Potter, to mourn his loss, and with him mother, sisters, wife and children. The balm that may assuage their grief must be found in reflecting upon his achievements, and upon the reward which he earned in his brief life.

---

[The Cincinnati Medical Journal, August, 1891.]

### DR. FRANK HAMILTON POTTER.

WE regret to be compelled to announce the death of Dr. Potter, as with it the profession loses one of its most able, conscientious and successful workers, and his place both in literature and practice will with difficulty be filled.

Dr. Potter was the only son and eldest child of Dr. Wm. Warren Potter, managing editor of the BUFFALO MEDICAL AND SURGICAL JOURNAL, and was associated with his father in the editorial management of this valuable publication.

He was born in Cowlesville, N. Y., January 8, 1860, and died July 16, 1891.

He graduated from the Buffalo Medical College in the class of 1882, at the early age of 22. Prior to his graduation he served in the Rochester City Hospital for two years.

He held the lectureship of descriptive anatomy and clinical assistant in Surgery in the Niagara University in 1883-4, and lecturer on botany in 1884-5, lecturer on materia medica from 1885 to 1888, and lecturer on laryngology from May, 1888, to 1891. In recognition of his efforts this University conferred upon him the *ad eundum* degree of M. D.

At the close of the session 1891, he severed his connection with this school and accepted the chair of clinical professor of laryngology in the Buffalo University Medical College.

A more fitting tribute cannot be paid to his memory than that of the

BUFFALO MEDICAL AND SURGICAL JOURNAL, when it says: "The profession join in the general sorrow pervading the community, and tender their sincere sympathy to the afflicted families for this strange dispensation which robs them of a devoted son and brother, a loving husband and father, and the city of a respected and honored citizen."

---

[From the Nashville Journal of Medicine and Surgery, August, 1891.]

We note, with extreme regret, the death of Dr. Frank H. Potter, of Buffalo, N. Y., at the age of 31. To Dr. William Warren Potter, his honored father, one of the editors of the BUFFALO MEDICAL AND SURGICAL JOURNAL, we extend our heartfelt sympathy.

---

[St. Joseph (Mo.) Medical Herald.]

### THE DEATH OF DR. FRANK H. POTTER.

The August number of the THE BUFFALO MEDICAL AND SURGICAL JOURNAL contains a notice of the death of this brilliant and promising young man, who gave evidence of a successful future. Already had he attained a high rank in his profession, and needed but time to make his mark among men. The loss of a promising child at any age is a severe affliction, which only those who have suffered a like loss can possibly appreciate or know the depth of sorrow and grief that overwhelm the heart of the parent; but when that child is a man, with a bright future before him, one who has shown the world what were his capabilities, the loss becomes oppressive and bears down the heart in the deepest suffering. But there is some alleviation of the parents' grief in the reflection and assurance that they have the sincere sympathy of the profession throughout the land. The editors of the *Herald* join in the expression, and offer to the bereaved their sincere condolence.

---

[From the Richmond (Va.) Practice, July, 1891.]

### DR. FRANK HAMILTON POTTER

The associate editor of the BUFFALO MEDICAL AND SURGICAL JOURNAL, and son of Dr. William W. Potter, of Buffalo, N. Y., died July 16th, after a short illness. He was a frequent contributor to medical literature. Personally, he was a most genial and courteous gentleman. Our sincere sympathy is extended to his bereaved family.

---

[From the North Carolina Medical Journal, July, 1891.]

Dr. Frank Hamilton Potter, of Buffalo, died on the 16th inst., as we learn from the *New York Medical Journal*. He was the son of Dr. William Warren Potter, editor of the BUFFALO MEDICAL JOURNAL, and was associated with his father in the editorship of that journal. Dr. Potter has our profound sympathy in his affliction.

[From the Alabama Medical and Surgical Age, August, 1891.]

### FRANK HAMILTON POTTER, M. D.

The sudden death of such a man as the late Dr. Frank Hamilton Potter, of Buffalo, N. Y., which occurred July 16th, in his 32d year, a man whose earlier student life already showed abundant fruits in practice, has brought anguish to his family, deep sorrow to his friends, and genuine regret to those that knew him even slightly.

Dr. Potter was born at Cowlesville, Wyoming county, New York, January 8, 1860, and was the only son of Dr. William Warren Potter, also a prominent member of the medical profession, residing in Buffalo.

The younger Dr. Potter graduated from the Buffalo Medical College in 1882, after having received academical training to fit him for his professional work. Prior to his graduation he served two years as resident physician in the Rochester City Hospital. He was engaged in general practice for several years, during which he served as assistant to the chair of surgery in Niagara Medical College, as lecturer on anatomy, and, finally, after adopting laryngology as his specialty, he was appointed lecturer on diseases of the nose and throat in that college. He served also as a member of the surgical staff of the Sisters of Charity and the Emergency hospitals. In 1889 he was elected secretary of the laryngological section of the American Medical Association, and lately had been chosen by the council for membership in the American Laryngological Association. Very recently the faculty of the medical department of the University of Buffalo elected him as clinical professor of laryngology in that institution. All these honors attest his general and special acquirements in his chosen field. He was twice appointed civil service commissioner, resigning in the midst of his second term because of the increase of professional cares. But he was always ready to give of his time and strength to the duties of the citizen. His was not a one-sided development. He was active, socially, as a member of the Saturn Club, and was one of the twenty-five members of the Thursday Club, a purely literary society.

No man who has worked thus, even though cut off untimely, has been here in vain. His influence in the community was good, and shall not cease. To his friends he has left a high example, and to those dearer ones a sweet, inspiring memory.

In June, 1887, he married Eva, daughter of Mr. L. G. Sellstedt, the well-known artist of Buffalo, and his widow and two children, one three years and the other four months old, survive him.

---

### ACTION OF THE AMERICAN LARYNGOLOGICAL ASSOCIATION.

The American Laryngological Association, at its August meeting, held in Washington, September 22 to 24, 1891, adopted the following memorial:

*Resolved*, That this association, recognizing the high intellectual and professional attainments of Dr. Frank H. Potter, deeply deplores that so bright a light should be put out at the very threshold of his professional career, and on the eve of his admission to this Society. That it tenders to his family its sincere sympathy in their bereavement, and mourns the loss with more than ordinary feelings of sorrow.

### ACTION OF THE CIVIL SERVICE REFORM ASSOCIATION.

The following resolution was adopted at a meeting of the executive committee of the Civil Service Reform Association, held September 8, 1891:

By the death of Dr. Frank H. Potter the Civil Service Reform Association of Buffalo loses from its executive committee an interested and active worker. His arduous service, without compensation, as a member of the municipal civil service commission, showed that as a physician he was keen to discern the causes of disease even in the body politic, and fearless in applying the needful remedies. Ashamed to satisfy his conscience with passive approval of what was good, or passive condemnation of what was bad, he gave to his city in full measure the energy and the intelligence which always characterized him. The city of Buffalo can ill afford to lose from its service, at the beginning of his usefulness, a citizen of such high mental and moral worth, and so unselfishly and actively interested in the cause of purer government.

FREDERIC ALMY,
*Secretary.*

